# Virological Predictors of Response to Retreatment in Hepatitis C Genotype 2 Infected Patients

**DOI:** 10.1371/journal.pone.0058882

**Published:** 2013-03-19

**Authors:** Chung-Feng Huang, Chia-Yen Dai, Ming-Lun Yeh, Jee-Fu Huang, Ching-I Huang, Ming-Yen Hsieh, Zu-Yau Lin, Shinn-Cherng Chen, Liang-Yen Wang, Suh-Hang Hank Juo, Wan-Long Chuang, Yi-Ching Lin, Ming-Lung Yu

**Affiliations:** 1 Institute of Clinical Medicine, College of Medicine, Kaohsiung Medical University, Kaohsiung, Taiwan; 2 Hepatobiliary Division, Department of Internal Medicine, Kaohsiung Medical University Hospital, Kaohsiung, Taiwan; 3 Department of Occupational Medicine, Kaohsiung Municipal Ta-Tung Hospital, Kaohsiung Medical University Hospital, Kaohsiung Medical University, Kaohsiung, Taiwan; 4 Department of Preventive Medicine, Kaohsiung Medical University Hospital, Kaohsiung, Taiwan; 5 Faculty of Internal Medicine, College of Medicine, Kaohsiung Medical University, Kaohsiung, Taiwan; 6 Department of Internal Medicine, Kaohsiung Municipal Hsiao-Kang Hospital, Kaohsiung Medical University Hospital, Kaohsiung, Taiwan; 7 Department of Internal Medicine, Kaohsiung Municipal Ta-Tung Hospital, Kaohsiung Medical University Hospital, Kaohsiung Medical University, Kaohsiung, Taiwan; 8 Department of Medical Genetics, Kaohsiung Medical University, Kaohsiung, Taiwan; 9 Department of Laboratory Medicine, Kaohsiung Medical University Hospital, Kaohsiung Medical University, Kaohsiung, Taiwan; University of Modena & Reggio Emilia, Italy

## Abstract

**Background/Aims:**

The impact of virological factors and interleukin-28B (IL-28B) genetic variants on retreatment of hepatitis C virus genotype 2 (HCV-2) treatment-experienced patients remains unknown.

**Methods:**

On-treatment virological responses and IL-28B rs8099917 genotype were determined in 46 HCV-2 treatment-experienced patients (42 previous relapsers; four previous non-responders) retreated with 24-week peginterferon/ribavirin.

**Results:**

Forty (87.0%) patients carried the rs8099917 TT genotype and 6 patients (13.0%) carried the TG/GG genotype. The sustained virological response (SVR; seronegativity of HCV RNA throughout 24 weeks of the post-treatment follow-up period) rate was 71.7%. Compared with previous non-responders, previous relapsers had a significantly higher SVR rate (78.6% vs. 0%, P = 0.004) and a lower relapse rate (17.5% vs. 100%, P = 0.04). All the previous non-responders were with the rs8099917 TT genotype. As for those who relapsed, treatment responses, including the rates of rapid virological response (RVR, 80.6% vs. 66.7%, P = 0.59), early virological response (EVR, 97.2% vs. 83.3%, P = 0.27), end-of-treatment virological response (97.2% vs. 83.3%, P = 0.27) and SVR (80.6% vs. 66.7%, P = 0.59) and relapse rate (17.1% vs. 20.0%, P = 1) did not differ significantly between patients with the rs8099917 TT and those with the non-TT genotype. Multivariate analysis revealed that the most important factor predictive of an SVR in the retreatment of HCV-2 was previous relapse; the only factor predictive of an SVR for previous relapsers was the achievement of an EVR. Compared with the achievement of a RVR, the attainment of an EVR was more accurate in predicting an SVR (88% vs. 74%).

**Conclusions:**

Peginterferon/ribavirin is effective in the retreatment of HCV-2 relapsers, especially among those who achieved an EVR.

## Introduction

Hepatitis C virus (HCV) infection is one of the leading threats to public health in Taiwan and worldwide.[1] Carriers might develop hepatocarcinogenesis and liver-related morbidity and mortality if left untreated. Successful HCV treatment using antiviral therapy would prevent the consequences of HCV infection.[2], [3] Recently, the introduction of direct antiviral agents (DAA) has greatly improved the sustained virological response (SVR) rate in patients with HCV genotype 1 (HCV-1) infection.[4] On the other hand, pegylated interferon (peginterferon)/ribavirin, which is the standard-of-care for HCV-2 patients, has provided satisfactory results with an SVR rate of 80–93%.[5]–[7] As a consequence, only a minority of HCV-2 patients fail antiviral therapy and are prone to be neglected because of the excellent treatment efficacy toward HCV-2 infection. It is noteworthy that these patients are still at high risk for liver disease progression and hepatocarcinogenesis.[2], [3], [8] Therefore, the issue of retreatment of HCV-2 patients who have failed previous treatments is important. However, it has rarely been addressed, which may reflect the limited number of patients in this specific scenario.

Recent studies have provided solid evidence that host interleukin 28B (IL-28B) genetic polymorphisms are associated with treatment efficacy in HCV-1 patients treated with peginterferon/ribavirin.[9]–[11] However, the application of DAAs seems to attenuate the role of host IL-28B genetic variants in HCV-1 infection.[12] On the other hand, results regarding the role of IL-28B in HCV-2 patients seem contradictory. Furthermore, the impact on viral kinetics was also inconsistent across studies. [13]–[15] A recent meta-analysis showed that favorable IL-28B polymorphisms were associated with a higher sustained virological response rate (SVR), but the effect was too limited to justify a clinical treatment decision.[16] It is noteworthy that the impact of host Il-28B genetic variants on HCV-2 treatment experienced patients with has never been explored. The current study aimed to elucidate the efficacy of HCV-2 patients retreated with 24-week peginterferon/ribavirin combination therapy. We also assessed the potential impact of host IL-28B genetic variants on a well-characterized Asian patient cohort.

## Methods

### Patients

Patients were recruited consecutively from one medical center and 2 regional core hospitals from 2002 to 2009 if they had relapsed (defined as HCV RNA seronegativity at the end of therapy but reappearance of viremia during follow-up) or if they were non-responders (defined as the presence of HCV RNA at the end of the prior course of therapy) to previous interferon-based therapy. The previous treatment course comprised conventional interferon at a dose of 3–6 million units thrice weekly or peginterferon alpha-2a (180 µg/week) or peginterferon alpha-2b (1.5 µg/kg/week) plus ribavirin for 24 weeks from the cohort that has been intervened previously.[13] Patients were excluded if they had any of the following: any other coexistent liver disorders (alcoholic liver disease, autoimmune hepatitis, primary biliary cirrhosis, sclerosing cholangitis, Wilson's disease and α_1_-antitrypsin deficiency); co-infection with hepatitis B or anti-human immunodeficiency virus; active use of illicit intravenous drugs; or a history of an uncontrolled psychiatric condition, pregnancy, decompensated cirrhosis or overt hepatic failure. All of the participants were retreated either with peginterferon alpha-2a (180 µg/week) or with peginterferon alpha-2b (1.5 µg/kg/week) plus weight-based ribavirin (1000 mg/d for a weight of <75 kg and 1200 mg/d for a weight of >75 kg) for 24 weeks. Serum HCV RNA was obtained using real-time polymerase chain reaction (RealTime HCV; Abbott Molecular, Des Plaines ILUSA, lower limit of quantitation <12 IU/mL) at baseline, treatment weeks 4 and 12, the end-of-treatment and 24 weeks after therapy.[17] All of the patients provided written informed consent before enrollment. The institutional review board at Kaohsiung Medical University Hospital approved the protocol, which conformed to the guidelines of the International Conference on Harmonization for Good Clinical Practice.

### Assessment of efficacy

A rapid virological response (RVR) was defined as seronegativity of HCV RNA at week 4 of treatment. An EVR was defined as a seronegative or at least a 2-log_10_ decrease from baseline in the serum HCV RNA at week 12 of treatment. An end-of-treatment virological response (EOTVR) was defined as seronegativity of HCV RNA at the end of treatment. The endpoint of the study was the achievement of a sustained virological response (SVR), which was defined as seronegativity of HCV RNA throughout 24 weeks of the post-treatment follow-up period.

### IL-28B genotyping and statistical analyses

Rs8099917 was selected as the candidate single-nucleotide polymorphism (SNP) in the current study. Genetic testing of IL-28B rs8099917 was determined using methods that have been previously described.[10], [13] The frequency was compared between groups using the χ^2^ test and a Yates correction or a Fisher exact test. Group means and standard deviations were compared using an analysis of variance test and the Student's *t* test or the Mann-Whitney U test. Serum HCV RNA levels were expressed after logarithmic transformation of the original values. The aspartate aminotransferase (AST)-to-platelet ratio index (APRI) was calculated using the following equation: (AST level/upper limit of normal range)/platelet counts (10^9^/L)×100. The APRI was used to reflect the severity of liver fibrosis.[18] The frequency of the rare allele (G) of the rs8099917 genotype was too low; thus, the rare homozygote (GG) and heterozygote (GT) genotypes were combined together when analyzing the SNP. To assess the relative contribution of the predictors of a RVR, a multivariable model was applied with age, sex, body weight, baseline HCV RNA levels, APRI, previous treatment regimen (optimal or suboptimal), previous virological response (relapse or non-response) and rs8099917 genotype as covariants. In addition to the above-mentioned variables, the achievement of a RVR and an EVR were taken into consideration while assessing determinants predictive of an SVR. The statistical analyses were performed using the SPSS 12.0 statistical package (SPSS, Chicago, IL, USA). All statistical analyses were based on two-sided hypothesis tests with a significance level of p<0.05.

## Results

### Patient profile

A total of 46 patients were recruited in this study. The basic demographic, virological, and clinical features of the patients were shown in table 1. Forty (87.0%) patients carried the rs8099917 TT genotype and 6 (13.0%) patients carried the rs8099917 TG/GG genotype. As for the previous virological response and treatment regimen, forty-two (91.3%) patients were previous relapsers and 4 (8.7%) patients were previous virological non-responders. The basic demographic, virological, and clinical features did not differ significantly between previous relapsers and non-responders. Fifteen (33%) patients previously received conventional interferon/ribavirin, and the remaining 31 (67%) patients received 24-week peginterferon/ribavirin combination therapy.

**Table 1:** Basic demographic, virological, and clinical features of HCV genotype 2 patients who failed interferon-based therapy.

	All patients (N = 46)	Relapsers (N = 42)	Non-responders (N = 4)	*P value*
Age, years, mean(SD)	58.6 (9.6)	58.6 (10.0)	58.0 (4.2)	0.90
Male, n (%)	25 (54.3)	23 (54.8)	2 (50.0)	1
Body weight, kg, mean (SD)	64.2 (8.6)	63.7 (8.9)	69.1 (1.7)	0.15
Baseline HCV RNA, log IU/ml, mean (SD)	5.45 (0.81)	5.39 (0.81)	5.99 (0.60)	0.16
Baseline HCV RNA > 400,000 IU/mL, n (%)	23 (50.0)	20 (47.6)	3 (75.0)	0.61
APRI, mean (SD)	2.05 (1.55)	1.99 (1.44)	2.60 (2.69)	0.46
AST, IU/l, mean (SD)	108.6 (73.9)	108.8 (75.9)	106.8 (57.3)	0.96
ALT, IU/l, mean (SD)	159.9 (130.6)	164.3 (135.5)	113.8 (41.3)	0.47
Rs8099917 TT genotype, n (%)	40 (87.0)	36 (85.7)	4 (100)	1
Previous optimal treatment regimen*, n (%)	31 (67.4)	28 (66.7)	3 (75.0)	1

Note: SD: standard deviation; AST: aspartate aminotransferase; ALT: alanine aminotransferase; APRI: aspartate aminotransferase-to-platelet ratio index. *defined as patients who had received 24 weeks of peginterferon/ribavirin

### Virological responses and factors associated with RVR and SVR

The rates of RVR, EVR, EOTVR, SVR and relapse were 76.1%, 93.5%, 91.3%, 71.7% and 21.4%, respectively. The rates of RVR (78.6% vs. 50.0%, P = 0.24) and EVR (95.2% vs. 75.0%, P = 0.24) were not significantly different between relapsers and non-responders. However, compared with non-responders, relapsers had significantly higher rates of EOTVR (95.2% vs. 50.0%, P = 0.03) and SVR (78.6% vs. 0%, P = 0.004) and a lower relapse rate (17.5% vs. 100%, P = 0.04). As shown in table 2, neither any baseline factor including host IL-28B genotype nor previous virological response was significantly associated with a higher RVR rate. Lower baseline HCV RNA levels, previous virological relapse and the achievement of a RVR and an EVR were factors that were significantly associated with an SVR in the univariate analysis (table 2). Multivariate analysis revealed that the only factor predictive of an SVR was previous relapse (table 3). Among patients who previously relapsed, the SVR rate was substantially higher in patients with an RVR and significantly higher in patients achieving an EVR compared with their counterparts (table 4). Multivariate analysis revealed that the only factor predictive of an SVR for previous relapsers was the achievement of an EVR (table 3).

**Table 2:** Univariate analysis of factors associated with rapid virological response and sustained virological response.

	RVR (+) (n = 35)	RVR (-) (n = 11)	*P* value	SVR (+)	SVR(-)	*P* value
				(n = 33)	(n = 13)	
Rs8099917 TT genotype, n (%)	31 (88.6)	9 (81.8)	0.62	29 (87.9)	11 (84.6)	1
Male sex, n (%)	20 (57.1)	5 (45.5)	0.50	18 (54.5)	7 (53.8)	0.97
Age (yrs, mean(SD))	57.9 (10.3)	60.7 (6.9)	0.40	57.7 (10.4)	60.9 (7.0)	0.32
Body weight, kg, mean (SD)	63.4 (9.0)	66.7 (7.2)	0.26	63.9 (8.6)	64.8 (9.0)	0.74
Baseline HCV RNA (log IU/ml, mean(SD))	5.37 (0.82)	5.70 (0.78)	0.24	5.29 (0.82)	5.84 (0.67)	0.04
Baseline HCV RNA > 400,000 IU/mL, n (%)	16 (45.7)	7 (63.6)	0.49	15 (45.5)	8 (61.5)	0.33
APRI, mean (SD)	2.02 (1.43)	2.14 (1.95)	0.83	2.04 (1.49)	2.05 (1.75)	0.98
AST (IU/l, mean (SD))	112.1 (78.0)	97.6 (61.1)	0.58	111.4 (78.6)	101.6 (62.8)	0.69
ALT (IU/l, mean (SD))	174.0 (141.0)	115.2 (78.9)	0.20	169.1 (141.3)	136.6 (99.5)	0.45
Virological responses of previous antiviral therapy, n (%)			0.24			0.004
Previous relapse	33 (94.3)	9 (81.8)		33 (100)	9 (69.2)	
Previous null responder	2 (5.7)	2 (18.2)		0 (0)	4 (30.8)	
Previous optimal treatment regimen*, n (%)	20 (57.1)	10 (90.9)	0.07	20 (60.6)	11 (84.6)	0.17
RVR (+), n (%)	-	-	-	28 (84.8)	7 (53.8)	0.05
EVR (+), n (%)	-	-	-	33 (100)	10 (76.9)	0.02

Note: SD: standard deviation; SVR: sustained virological response; RVR: rapid virological response; EVR, early virological response. AST: aspartate aminotransferase; ALT: alanine aminotransferase; APRI: aspartate aminotransferase-to-platelet ratio index.* defined as patients who had received 24 weeks of peginterferon/ribavirin

**Table 3:** Logistic regression analysis of factors associated with sustained virological response in all patients and relapsers.

Variables	Odds Ratio	95% Confidence Intervals	*P* value
***All patients***			
Relapsers	1		
Non-responders	0.000		<0.0001
***Relapsers***			
EVR	1		
Non-EVR	0.000		<0.0001

Note: EVR: early virological response.

**Table 4:** Univariate analysis of factors associated with sustained virological response in previous relapsers.

	SVR (+) (n = 33)	SVR(-) (n = 9)	*P* value
Rs8099917 TT genotype, n (%)	29 (87.9)	7 (77.8)	0.59
Male sex, n (%)	18 (54.5)	5 (55.6)	1
Age (yrs, mean(SD))	57.7 (10.4)	62.1 (7.8)	0.24
Body weight, kg, mean (SD)	63.9 (8.6)	62.9 (10.4)	0.78
Baseline HCV RNA (log IU/ml, mean(SD))	5.29 (0.82)	5.78 (0.73)	0.11
Baseline HCV RNA > 400,000 IU/mL, n (%)	15 (45.5)	5 (55.6)	0.71
APRI, mean (SD)	2.04 (1.49)	1.81 (1.29)	0.67
AST (IU/l, mean (SD))	111.4 (78.6)	99.3 (68.4)	0.68
ALT (IU/l, mean (SD))	169.1(141.3)	146.8 (117.6)	0.67
Previous optimal treatment regimen*, n (%)	20 (60.6)	8 (88.9)	0.23
RVR (+), n (%)	28 (84.8)	5 (55.6)	0.08
EVR (+), n (%)	33 (100)	7 (77.8)	0.04

Note: SD: standard deviation; SVR: sustained virological response; RVR: rapid virological response; EVR, early virological response. AST: aspartate aminotransferase; ALT: alanine aminotransferase; APRI: aspartate aminotransferase-to-platelet ratio index.* defined as patients who had received 24 weeks of peginterferon/ribavirin.

### On-treatment responses in predicting treatment outcome

Because the achievement of an EVR, but not a RVR, was independently predictive of treatment outcome among relapsers, we further analyzed the accuracy of the two on-treatment factors in predicting an SVR. As shown in table 5, the achievement of an EVR provided a negative predictive value of 100%. The positive predictive value for an SVR was similar between patients with a RVR (80% for all patients and 85% for relapsers) and an EVR (77% for all patients and 83% for relapsers). The achievement of an EVR provided a better accuracy in predicting an SVR (88% for all patients and 83% for relapsers) compared to the factor of a RVR (74% for all patients and 75% for relapsers).

**Table 5:** Accuracy of the achievement of a RVR and an EVR in predicting an SVR.

On treatment response	SVR(+)	SVR(-)	*P value*	SEN	SPE	PPV	NPV	ACC
	n (%)	n (%)		%	%	%	%	%
***All patients***	n = 33	n = 13						
RVR (+)	28 (85)	7 (54)	0.05	85	46	80	55	74
EVR (+)	33 (100)	10 (77)	0.02	100	23	77	100	88
***Previous Relapsers***	n = 33	n = 9						
RVR (+)	28 (85)	5 (56)	0.08	85	44	85	44	75
EVR (+)	33 (100)	7 (78)	0.04	100	22	83	100	83
***Previous Non-responders***	n = 0	n = 4						
RVR (+)	-	2 (50)	-	-	50	-	100	-
EVR (+)	-	3 (75)	-	-	25	-	100	-

Note: SVR: sustained virological response; RVR: rapid virological response; EVR, early virological response

### Influence of IL-28B genetic variants on treatment responses

**Figure 1:**
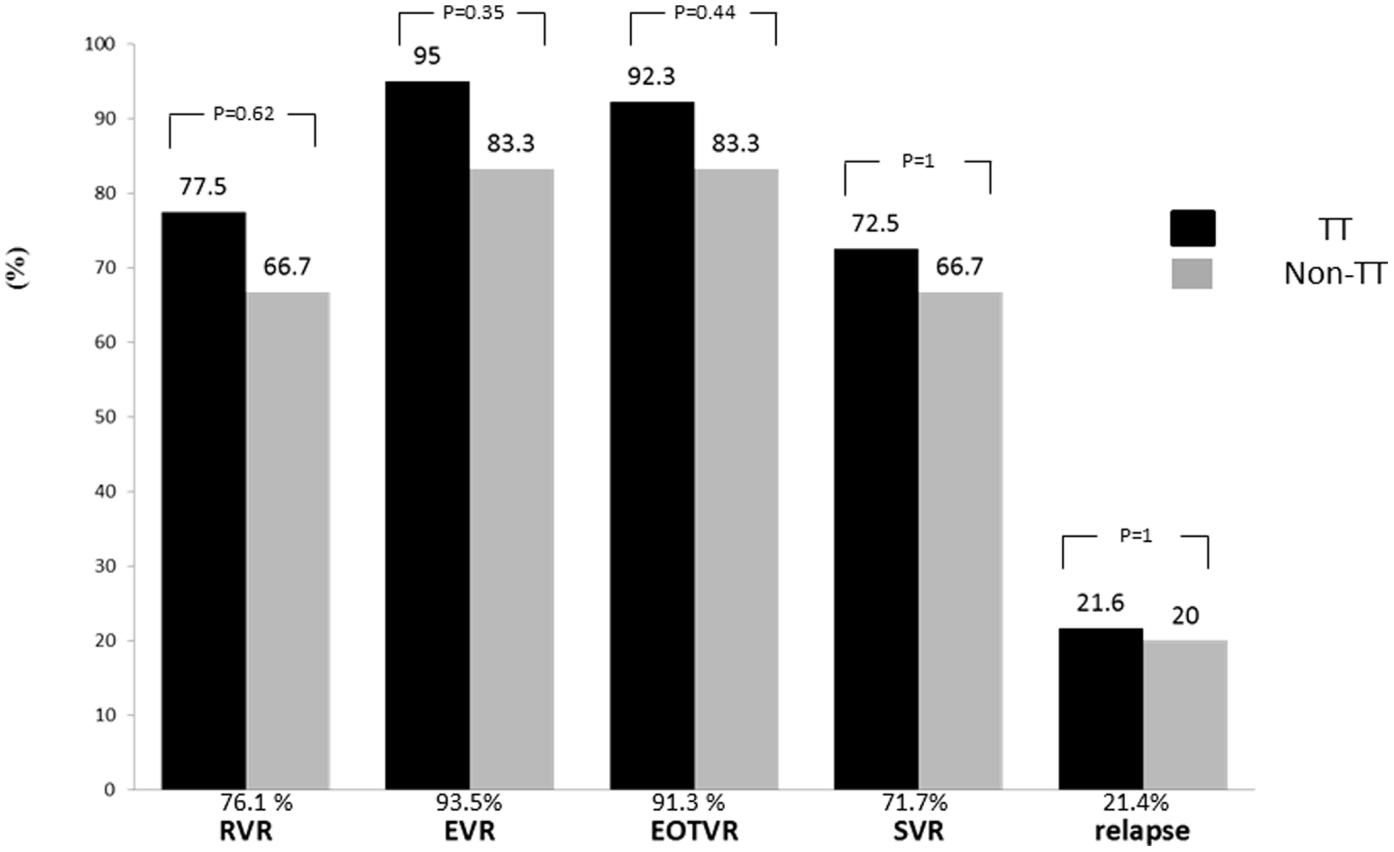
Treatment responses between patients with different rs8099917 genotypes. Black bar represents patients with rs8099917 TT genotype. Brown bar represents patients with rs8099917 GT/GG genotype. RVR, rapid virological response. EVR, early virological response. EOTVR, end of treatment virological response. SVR, sustained virological response.

Treatment responses including rates of RVR (77.5% vs. 66.7%, P = 0.62), EVR (95.0% vs. 83.3%, P = 0.35), EOTVR (92.3% vs. 83.3%, P = 0.44) and SVR (72.5% vs. 66.7%, P = 1) and relapse rate (21.6% vs. 20.0%, P = 1) were similar between patients with the TT or non-TT genotype (fig 1). All the 4 non-responders were with rs8099917 TT genotype. Treatment responses including rates of RVR (80.6% vs. 66.7%, P = 0.59), EVR (97.2% vs. 83.3%, P = 0.27), EOTVR (97.2% vs. 83.3%, P = 0.27) and SVR (80.6% vs. 66.7%, P = 0.59) and relapse rate (17.1% vs. 20.0%, P = 1) did not differ significantly between previous relapsers with rs8099917 TT or non-TT genotype.

## Discussion

In the era of DAA, 24 weeks of pegylated interferon plus ribavirin treatment remains the standard of care for HCV-2 infected patients [19], [20] with an SVR rate of 90%. [5], [21] As a result, studies involving HCV-2 treatment experienced patients with were scarce, and majority of the results were linked to other HCV genotypes as a subgroup analysis.[22]-[28] To the best of our knowledge, this is the first report that focused on previously treated patients with HCV-2 and explored the possible role of viral factors and host IL-28B genetic polymorphisms in the retreatment outcome. We noted that previous virological response was the most critical factor predictive of treatment success. Four-fifths of the patients who previously relapsed, but none of the non-responders, had an SVR after 24 weeks of peginterferon/ribavirin retreatment. On treatment viral kinetics rather than host IL-28B genetic variants might play a role in determining an SVR in relapsers.

Although only a minority of HCV-2 patients failed to eradicate virus after 24 weeks of standard-of-care, these patients remained at a great risk of liver disease progression.[2], [29] However, standard strategies toward treatment-experienced HCV-2 patients has not been clearly setup. Discordant results regarding HCV-1 retreatment by interferon-based therapy have been observed across studies.[30] Similarly, an SVR rate of ranging from 31% to 79% has been reported in small-scale studies of HCV-2 retreatment (table 6). [22]–[28] The divergent reports might be attributed to the limited sample size and the diverse patient characteristics. A variety of previous and current treatment regimens might also account for the inconclusive results. Basso et al.[23] has reported an SVR rate of 78.6% in previous relapsers, in which study patients who failed to eradicate HCV infection by conventional interferon (3 MU thrice weekly)/ribavirin were retreated with suboptimal pegylated interferon alpha-2b (1 µg/kg/week) plus ribavirin (800–1200 mg/day) for 24 weeks. In the current study, we demonstrated a similar SVR rate for previous relapsers retreated with 24 weeks of peginterferon/weight-based ribavirin. The treatment efficacy remained consistent irrespective of the previous treatment regimen. The finding might provide more insight for clinicians in daily practice because pegylated interferon/ribavirin has been the standard of care for a decade, and most of the HCV-2 treatment experienced patients nowadays might have received an optimal regimen previously. It raised the issue that extending therapy to 48 weeks might be beneficial for certain treatment naïve patients with unfavorable early viral kinetics and researches that identify the potential candidates for prolonged treatment remain elusive.[19]

**Table 6:** Studies regarding HCV genotype 2 retreatment with pegylated interferon plus ribavirin.

	Case No	Regimen	Previous virologicalresponse	SVR rate	Reference
Shiffman et al., 2004	31	pegylated interferon alfa-2a (180 µg/week) plus ribavirin (1000–1200 mg/day) for 48 weeks	non-responders with advanced fibrosis	65%	[24]
Jacobson et al.,2005	26*	pegylated interferon alfa-2b (1.0–1.5 µg/kg/week) plus ribavirin (800–1200 mg/day) for 48 weeks	relapsers and non-responders	31% (non-responder: 5%)	[25]
Krawitt et al., 2005	24	pegylated interferon alfa-2b (100–150 µg/week) plus ribavirin (1000 mg/day) for 48 weeks	relapsers (n = 17) and non-responder (n = 7)	relapsers:59%; non-responders:57%	[26]
Basso et al.,2007.	28*	pegylated interferon alfa-2b (1 µg/kg/week) plus ribavirin (800–1200 mg/day) for 24 weeks	relapsers	78.6%	[23]
Jensen et al.,2009	7	pegylated interferon alfa-2a (360 µg/wk for 12 weeks, then 180 µg/wk for 36–60 weeks or 180 µg/wk for 48–72 weeks) plus ribavirin (800–1200 mg/day)	Non-responders	N/A	[27]
Poynard et al., 2009	75	peginterferon alfa-2b (1.5 µg/kg/wk) plus weight-based ribavirin (800–1400 mg/day); treatment duration varied according to week 12 response	relapsers and non-responders with METAVIR score ≥2	relapsers:61%; non-responders:46%	[28]
Oze et al.,2011	18	pegylated interferon alfa-2a (180 µg/week) or alfa-2b (1.5 µg/kg/week) plus ribavirin (800–1200 mg/day) for 24 weeks	relapsers (n = 17) and non-responder (n = 1)	56%	[22]

Note: *including hepatitis C virus genotype 2 and 3

A common finding in studies regarding HCV-1 retreatment was that compared with previous relapsers, non-responders had significantly worse retreatment outcomes.[30] However, this was not always the case in HCV-2 studies. Jacobson et al. [25] reported an SVR of 5% for non-responders who received 48 weeks of pegylated interferon alpha-2b (1.0–1.5 µg/kg/week) plus ribavirin (800–1200 mg/day) combination therapy. On the contrary, an SVR rate of 57–65% for non-responders has been reported by other studies.[26], [28] None of the non-responders in our study had an SVR. However, the sample size was too small for the results to be conclusive. Recently developed DAAs have become the standard of care for HCV-1 infection.[4] This innovation, in conjunction with peginterferon and ribavirin, substantially improved the treatment efficacy in treatment-naïve and -experienced HCV-1 patients. Nevertheless, the development of small molecules against HCV-2/3 remains in its early stages. [31], [32] The strategy of extending the retreatment duration [24], [26] or applying DAAs to the difficult-to-treat population on the basis of cost-effectiveness [33] requires further exploration.

Emerging data have demonstrated that favorable host IL-28B genetic variants have been associated with a higher SVR rate in HCV-1 patients.[9]–[11] In contrast, results regarding the role of IL-28B in HCV-2 patients were conflicting.[13]–[15] A recent meta-analysis has shown that favorable IL-28B polymorphisms increase the SVR rate by 5%, but the predictive value was limited compared to other predictive factors.[16] In addition, the impact of IL-28B on the retreatment of HCV-2 infection has never been explored. We have demonstrated that IL-28B genetic variants would not determine either early viral kinetics or final treatment outcome in HCV-2 treatment-experienced patients. On the other hand, better on-treatment responses might be associated with a higher SVR rate. The achievement of a RVR has been suggested to be the most important factor predictive for an SVR regardless of host IL-28B genetic variants in HCV-1 infection [10], [11] and is the most critical factor for HCV-2 naïve patients [5]. However, the SVR rate was not significantly different in patients with or without a RVR, and the achievement of an EVR was more accurate for retreated patients. The limited number of cases might partly account for the results in this study. However, it should be noted that viral elements to interferon responsiveness [34], as well as the host-virus interaction, might have been altered in the treatment experienced patients. The viral kinetics of interferon-based therapy in treatment experienced patients might be different from treatment-naïve patients. Whether the week 12 rather than week 4 responsiveness is a better surrogate for predicting an SVR for this special population deserves further investigation.[27], [28] One limitation of this current study includes the limited number of cases, which render our findings less conclusive, particularly for the previous non-responders. Furthermore, our results have not been validated in other ethnic groups with different IL-28B genotypes. However, the role of IL-28B genetic testing was fully explored in the current study, and the satisfactory outcomes with peginterferon/ribavirin in patients who relapsed raised the issue of the cost-effectiveness of DAAs, which would be especially important in areas where HCV-2 infection is endemic.[1], [21]

In conclusion, peginterferon/ribavirin is effective in the retreatment of HCV-2 relapsers, particularly among those who achieved an EVR. Host IL-28B genetic variants might play a minimal role in HCV-2 treatment-experienced patients. The role of DAAs in interferon-resistant HCV-2 patients awaits further elucidation.
